# Performing whole-genome association analysis of winter wheat plant height using the 55K chip

**DOI:** 10.3389/fpls.2024.1471636

**Published:** 2025-01-17

**Authors:** Yindeng Ding, Guiqiang Fan, Yonghong Gao, Tianrong Huang, Anding Zhou, Shan Yu, Lianjia Zhao, Xiaolei Shi, Sunlei Ding, Jiahao Hao, Wei Wang, Jikun Song, Na Sun, Hui Fang

**Affiliations:** ^1^ Institute of Grain Crops, Xinjiang Academy of Agricultural Sciences, Xinjiang, China; ^2^ College of Agriculture, Xinjiang Agricultural University, Xinjiang, China; ^3^ Institute of Crop Variety Resources, Xinjiang Academy of Agricultural Sciences, Xinjiang, China; ^4^ Department of Computer Science and Information Engineering, Anyang Institute of Technology, Anyang, China; ^5^ Cotton Research Institute, Chinese Academy of Agricultural Sciences, Anyang, China; ^6^ Yili Prefecture Institute of Agricultural Sciences/Yili Prefecture Key Laboratory of Crop Breeding and Quality Detection, Yining, China

**Keywords:** winter wheat, plant height, 55K SNP array, GWAS, candidate genes

## Abstract

Plant height is a critical agronomic that affects both plant architecture and yield. To decipher the genetic mechanisms underlying winter wheat plant height and identify candidate genes associated with this trait, we conducted phenotypic analysis on 239 wheat varieties (lines) collected from around the world. This analysis was complemented by genotyping using the wheat 55K SNP chip. A Wholegenome association analysis (GWAS) of wheat plant height was conducted utilizing the MLM (Q+K) model within TASSLE software. The results revealed significant phenotypic variation in wheat plant height across different years, with coefficients of variation ranging from 0.96% to 1.97%. Additionally, there was a strong correlation in plant height measurements between different years. GWAS identified 44 SNP markers significantly associated with wheat plant height across various environments (P ≤ 0.00001), predominantly distributed on chromosomes 1B, 1D, 2A, 2B, 2D, 3B, 3D, 4A, 4B, 6B, 6D, and 7D, explaining individual phenotypic variance rates ranging from 5.00% to 11.11%. Further, by mining association loci with substantial phenotypic effects and stability across multiple environments, seven candidate genes related to wheat plant height have been identified. This study provides new genetic markers and resources for improving wheat plant height.

## Introduction

As a highly adaptable and widely grown food crop in the world, wheat provides about 21 percent of food calories and 20 percent of protein for humans (Maccaferri et al., 2014). Its high nutritional value and unique gluten properties permit the preparation of a wide variety of food products, therefore it has become one of the most important food crops (Wang et al., 2022; Liu et al., 2023). The key goal of their breeding has been to increase yield (Shiferaw et al., 2013; Jin et al., 2019; Wang et al., 2024). Wheat plant height is a key agronomic trait affecting plant conformation and yield. As an aboveground support organ, the wheat stem connects various physiological activities between the leaves and the grain. If wheat plant height is too high, it can lead to plant collapse, which directly reduces yield, while too short a plant can lead to overcrowding of canopy leaves, which affects photosynthetic rate, thus making biomass unable to meet the demand, which in turn reduces yield (Wang and Li, 2008; Godin, 2000). Plant height is simultaneously regulated by both genes and the environment (Li et al., 2020). Dwarf wheat has dramatically increased wheat yields in practice due to its strong resistance to felling (Miao et al., 2022). About 30 dwarf genes have been reported, mainly on chromosomes 2A, 2B, 2D, 3B, 4B, 4D, 5A, 6A, 7A, and 7B, of which only the dwarf genes Rht1, Rht2, Rht8, Rht11, Rht13, and Rht24 have been applied in wheat breeding both at home and abroad, and have affected agronomic traits to varying extents to improve yields (Sun et al., 2017). Throughout the world, 70% of wheat varieties contain Rhtl or Rht2. In the Huanghuai wheat region of China, in addition to Rhtl and Rht2, a few short-stalked genes, such as Rht8 and Rht24, have no significant negative effect on yield traits while reducing plant height, and have been selected for use in breeding (Li et al., 2018; Liu et al., 2017). The discovery and utilization of new excellent dwarf genes not only can enrich the genetic resources of existing dwarf genes, but also is an important basis for the sustainable development of wheat highyield breeding. It is of great significance to analyse the genetic effect of plant height and to explore the functional genes related to plant height for the improvement of wheat varieties.

To the present, many scholars at home and abroad have conducted QTL localization studies on wheat plant height using linkage analysis and association analysis methods, and identified a large number of primary QTL loci for controlling plant height. Through QTL chain localization, Gao et al. (2015) used a population of recombinant inbred lines (RILs) constructed with ‘Zhou 8425’ and ‘China Spring’ as the parents as the material to localize six stable QTLs related to plant height, and a single QTL could explain 2.3%-33.2% of the A single QTL could explain 2.3%-33.2% of the phenotypic variation, among which two QTLs were colocalized with the short culm genes Rhtl and Rht2, respectively. Xiong et al. (2021) utilized a population of RILs constructed from an early tasselling mutant (eh1) and ‘Rotary Selection 987’ to identify seven QTLs located on chromosomes 2A, 4A, 4B, and 6B for controlling the height of wheat plants, with phenotypic variance contributions ranging from 5.5% to 34.4%. Li et al. (2020) obtained four QTLs controlling plant height and predicted four candidate genes encoding IAAamino acid hydrolase ILR1, growth regulator, flowering promoter, and phosphosucrose synthase in a population of RILs constructed from ‘Semimanzan’ and ‘Jeimai 22’. Li et al. (2020) identified three stable QTLs tightly linked to plant height using two recombinant inbred line (RIL) populations. They developed competitive allelespecific PCR (kompetitive allelespecific PCR, KASP) markers for OSE-Liscau-2CN-5A within this interval. Three candidate genes, all encoding cytochrome P450 and highly expressed in wheat stems, were discovered in this region. Qu et al. (2021) identified 21 QTLs closely linked to plant height in three populations of RILs, and identified a gene encoding a gibberellinregulated protein in the qPH-4D-1 interval of chromosome 4D, which is related to the regulation of plant height in wheat. The association analysis has been widely used in the genetic analysis of wheat plant height. Sukumaran et al. (2015) conducted a genome-wide association study (GWAS) on 287 highyielding wheat varieties, identifying two significant SNP loci associated with plant height. These loci were located on chromosomes 1A and 6A, contributing 6% and 7% of the phenotypic variance, respectively. Sun et al. (2017) conducted a genome-wide association study (GWAS) using SNP markers and 163 common wheat varieties from the Huanghuai region, identifying a total of 143 significant loci associated with wheat plant height. These loci were distributed across chromosomes 1B, 2A, 2B, 2D, 4B, 4D, and 6D, explaining 35.8% to 65.6% of the phenotypic variance. Rabbi et al. (2021), on the other hand, performed GWAS analysis on 361 US wheat varieties, detecting 20 significant SNP loci associated with plant height. These loci explained 4.54% to 48.01% of the phenotypic variance and predicted four candidate genes related to plant height on chromosomes 2A, 3A, and 6A.

Although some progress has been made in the basic research on plant height genetics, due to the large wheat genome and the complexity of the quantitative genetic mechanism of plant height, most of the genetic variation cannot be directly applied to molecular breeding, and its genetic analysis needs to be further expanded and deepened. Therefore, in this study, 239 wheat varieties (lines) were used as materials, and 16649 SNP markers covering the whole genome were used for genomewide association analysis of plant height in four environments, to detect the significant association loci and discover the relevant candidate genes, with a view to providing theoretical basis for the molecular breeding of plant height in wheat, and the excavation and utilisation of the candidate genes.

## Materials and methods

### Materials

A total of 239 natural populations consisting of wheat breeding varieties and foreign introduced varieties promoted in winter wheat areas of China were used as test materials. Of these, 213 were from China, including 6 from Anhui, 34 from Beijing, 19 from Hebei, 19 from Henan, 5 from Jiangsu, 11 from Shandong, 1 from Shanxi, 3 from Shaanxi, 1 from Tianjin and 115 from Xinjiang. There were 26 foreign materials, including 25 from the USA and 1 from Ukraine. All these materials could grow and develop normally in the test site.

### Multi-environment trials

The experiments were conducted from September 2019 to July 2022 at the ZePu Breeding base of Xinjiang Academy of Agricultural Sciences (77°16’17.22”N, 38°11’21.65”E), and in June 2021 at the Anningqu base of Xinjiang Academy of Agricultural Sciences (43°58’53.38”N, 87°30’17.72”E). The ZePu region exhibits a continental warm-temperate arid climate characterized by low annual precipitation. Overall, rainfall levels remain minimal, consistent with the arid nature of the Xinjiang region. The area experiences distinct four seasons, marked by significant diurnal temperature variations, with summers being notably warm and winters relatively cold. The Anningqu area is characterized by a temperate continental climate, with perennial low precipitation levels primarily concentrated during the summer months of June to August. Spring (March to May) experiences a gradual rebound in temperatures, although the rate of warming is relatively slow. In summer (July), the region is influenced by subtropical high-pressure systems, resulting in hot weather. Autumn (September to October) sees a decrease in rainfall alongside a gradual decline in temperatures. Winter (November to March) is dominated by prevailing northwesterly winds, leading to cold conditions, with snowfall being a prominent feature. During this season, temperatures can drop significantly, reaching as low as -20°C. In these trials, ZePu in Xinjiang served as E1 in 2020, E2 in 2021, and E4 in 2022, while Anningqu in Xinjiang was E3 in 2021. The best linear unbiased prediction (BLUP) of value were obtained using R 4.0 (the Matrix and lme4 packages). Each environment point utilized a randomized complete block design over three years. Each plot consisted of two rows, each 1 meter long and spaced 0.2 meters apart, with a sowing density of 350,000 seeds per hectare, oriented in a north-south direction. Standard field management practices including light, water, fertilizer, and other agronomic practices were followed consistently across the three replicates. The cultivation and production conditions were identical for each replicate.

### Measurement of plant height

After the milky stage of wheat, a straightedge was purchased to measure the length from the ground to the top of the spike (excluding the awns) in cm. 5 plants with similar growth were measured in each plot, and the average was taken as the height of the plant in the plot (**Supplementary Table S2**).

### Statistical analysis

The process of phenotyping was mainly ANOVA as well as descriptive statistics of phenotypes, significance test of difference and correlation analysis, in addition all statistical analyses of data were implemented in IDE Spyder under Anacondas3, using Python 3.8.8 for data processing, on a computer with an Intel i7-6800 K 3.40 GHz CPU, 16 GB of RAM, and Nvidia GeForce GTX 2080Ti on a graphics workstation running the Win10 operating system. Statistical analyses, correlation analyses and significance of difference tests for wheat plant height were performed using the application software Pandas 1.3.2, Matplotlib 3.4.2, Scikit-Learn 0.24.2 and SPSS 21.0.

### GWAS analysis

A total of 16649 SNP markers were identified and used for subsequent analysis as previously described by Ding et al. (2023) (**Supplementary Table S3**). The plant height (PH) in the four different environments were used for GWAS analysis. A previous study analysed the population structure and linkage disequilibrium (LD). The mixed linear model (MLM) in TASSEL 5.0 was used for GWAS, and the population structure was considered as a fixed effect (Ding et al., 2023). The Bonferroni threshold of SNP significance was P ≤ 0.00001. Manhattan plots were generated by using the “CMplot” package for R (Zhu et al., 2008).

## Results

### Analysis of phenotypic variation of plant height

**Figure 1:**
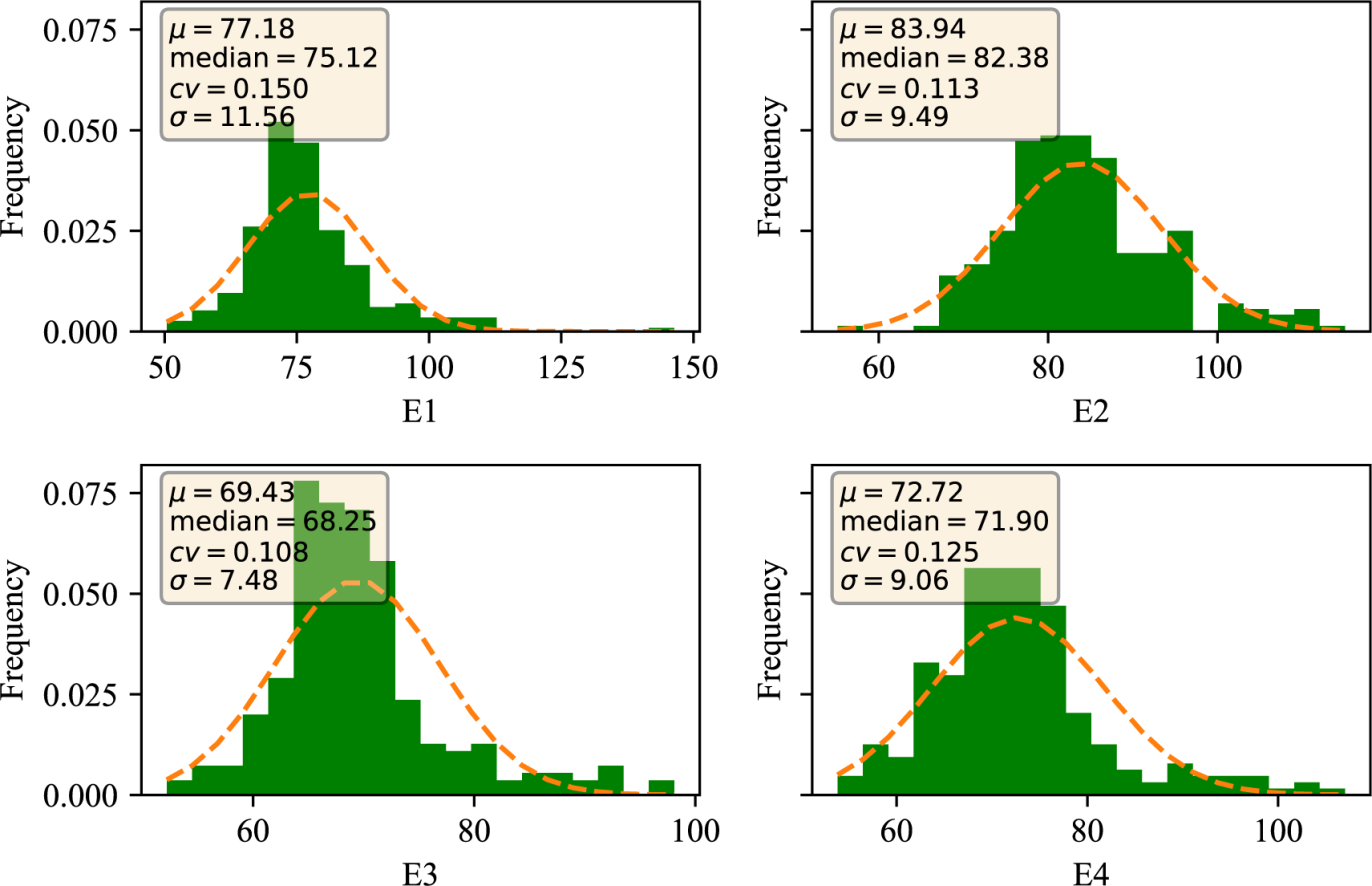
Distribution of traits of wheat plant height in different environments. E1: 2020 Zepu, Xinjiang; E2: 2021 Zepu, Xinjiang; E3: 2021 Anning Drain, Xinjiang E4: 2022 Zepu, Xinjiang.

Wheat plant height was phenotyped at four environmental sites, E1, E2, E3, and E4. The data were evaluated through four dimensions: mean (denoted by μ), median (denoted by median), coefficient of variation (denoted by cv), and standard deviation (denoted by σ). As seen in **Figure 1**, the mean μ of plant height in the E1 environment was 77.18, the median was 75.12, the coefficient of variation was 15.0%, and the standard deviation was 11.56; the mean μ of plant height in the E2 environment was 83.94, the median was 82.38, and the coefficient of variation was 11.3%, and the standard deviation was 9.49; and the mean μ of the germination-twigging stage in the E3 environment was 69.43, the median was 68.25, coefficient of variation was 10.80%, and standard deviation was 7.48; the mean μ of plant height under E4 environment was 72.72, median was 71.90, coefficient of variation was 12.50%, and standard deviation was 9.08 (**Table 1**). The ANOVA indicated that the environment and genotype interaction had significant effects on PH (*P* < 0.01) (**Supplementary Table S1**). Overall, the data under different environments showed continuous and normal distribution, which was in line with the characteristics of typical quantitative traits. These results provide reliable phenotypic data for the genetic analysis of PH.

**Table 1:** Descriptive statistics of wheat plant height.

Envs	Min (cm)	Max (cm)	Average (cm)	Standard Devition	Coefficient of Variation
E1	50.41	146.38	77.18	11.59	15.00%
E2	55.13	115.06	83.94	9.51	11.30%
E3	52.20	98.10	69.43	7.50	10.80%
E4	53.81	107.02	72.72	9.08	12.50%

### Correlation analysis of plant height

**Figure 2:**
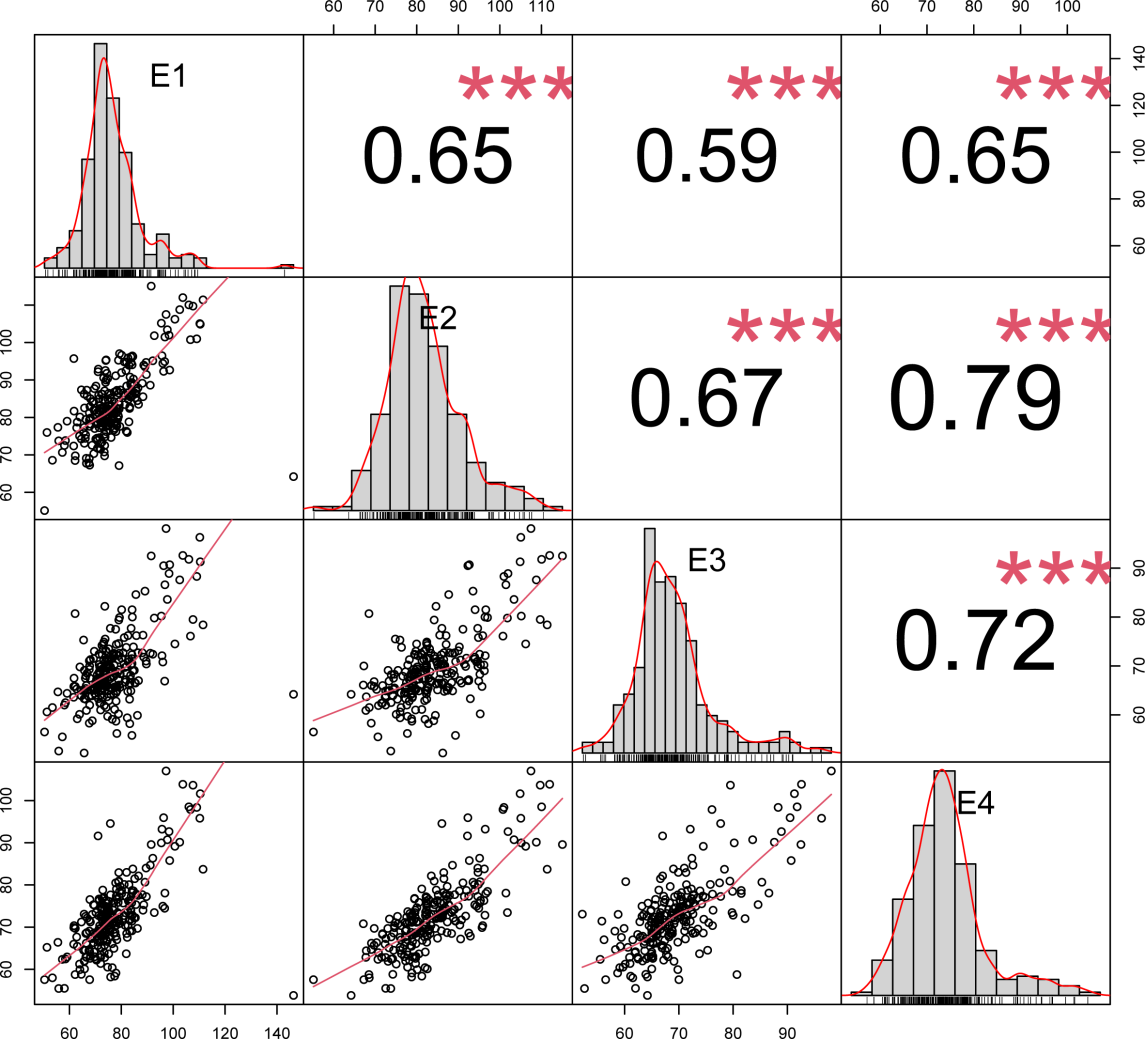
Correlation plot of wheat plant height in different environments.

The results of correlation analyses of plant height at four environmental points, E1, E2, E3 and E4, are shown in **Figure 2**. The figure compares the correlations of plant height in winter wheat under different environments, and the results show that the correlations were significant (p<0.001) in most cases. Specifically, the correlations of plant height ranged from -0.59 to -0.65 in E1, 0.65 to 0.79 in E2, 0.59 to 0.72 in E3, and 0.65 to 0.79 in E4. Taken together, the range of correlations of plant height in different environments was from 0.59 to 0.79, which were all significant (p<0.001).

### GWAS analysis of plant height

**Figure 3:**
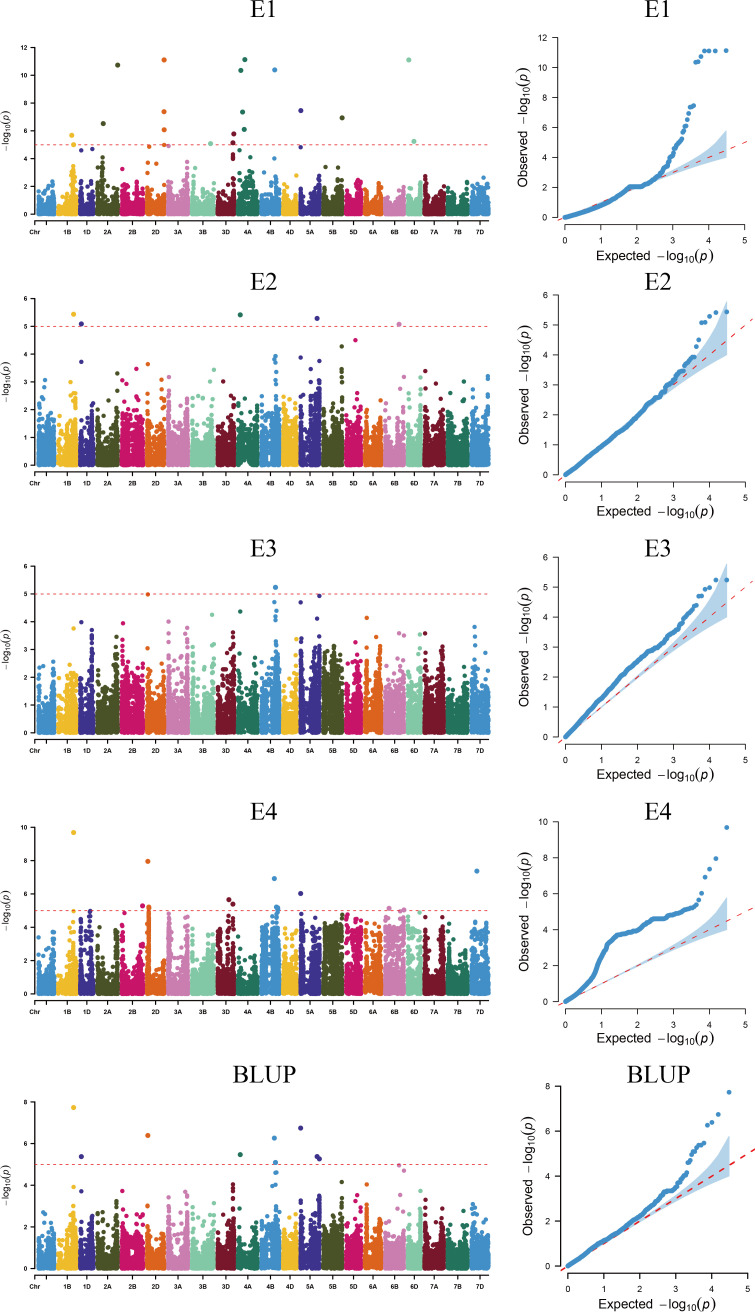
Manhattan plot of plant height in different environments.

Using TASSEL 5.0 software, a genome-wide association analysis was conducted on 239 wheat varieties (lines) using 16,649 high-quality SNP markers identified from a 55K SNP chip and combined with SPAD content measurements. The MLM (Q+K) mod-el was employed, with markers considered significantly associated with traits when (P ≤ 0.00001). Markers detected across multiple environments were considered stable genetic loci (**Figure 3**, **Table 2**). The GWAS results revealed a total of 44 markers associated with plant height, distributed across chromosomes 1B, 1D, 2A, 2B, 2D, 3B, 3D, 4A, 4B, 6B, 6D, and 7D. Individual markers explained phenotypic variance rates ranging from 5.00% to 11.11%. Notably, AX-110425403 located on chromosome 1B was detected in environments E1, E2, E3 and BLUP, with phenotypic variance rates ranging from 5.00% to 9.68%. AX-108850618 located on chromosome 1D was detected in environments E2 BLUP, with phenotypic variance rates ranging from 5.09% to 5.37%. AX-94748732 on chromosome 3D was detected in environments E1,E4 and BLUP, with phenotypic variance rates of 4.04% and 5.39%, respectively. AX-109306628 on chromosome 4A was detected in environments E2 and BLUP, with phenotypic variance rates of 5.41% and 5.47%, respectively. AX-109392949 on chromosome 4B was detected in environments E4 and BLUP, with phenotypic variance rates of 6.29% and 6.95%, respectively. AX-111046577 on chromosome 4B was detected in environments E3 and BLUP, with phenotypic variance rates of 5.09% and 5.24%, respectively. AX-111230804 on chromosome 4B was detected in environments E3 and BLUP, with phenotypic variance rates of 5.09% and 5.24%, respectively. AX-89577308 on chromosome 4B was detected in environments E4 and BLUP, with phenotypic variance rates of 4.61% and 5.21%, respectively. AX-109934491 on chromosome 5A was detected in environments E2 and BLUP, with phenotypic variance rates of 5.29% and 5.38%, respectively.AX-110644627 on chromosome 5A was detected in environments E4 and BLUP, with phenotypic variance rates of 6.03% and 6.74%, respectively. AX-110616775 on chromosome 6B was detected in environments E4 and BLUP, with phenotypic variance rates of 4.70% and 5.04%, respectively. AX-111593090 on chromosome 6B was detected in environments E2 and BLUP, with phenotypic variance rates of 4.96% and 5.07%, respectively. Other markers were detected in single environments.

**Table 2:** Information of significantly associated loci for wheat plant height.

Marker	Chr.	Position (Mb)	P-value	*R^2^* (%)	Environment
AX-110425403	1B	581.166658	9.9313E-06	5.00	E1
AX-110425403	1B	581.166658	3.6708E-06	5.44	E2
AX-110425403	1B	581.166658	2.0747E-10	9.68	E4
AX-110425403	1B	581.166658	1.86E-08	7.73	BLUP
AX-111228420	1B	512.31147	2.0967E-06	5.68	E1
AX-108850618	1D	42.612293	8.1813E-06	5.09	E2
AX-108850618	1D	42.612293	4.22E-06	5.37	BLUP
AX-109472712	2A	767.939996	1.851E-11	10.73	E1
AX-110472753	2A	227.266911	3.0102E-07	6.52	E1
AX-109852602	2B	778.014268	0.000005194	5.28	E4
AX-108764406	2D	641.122338	4.1945E-08	7.38	E1
AX-109462841	2D	71.768788	6.1277E-06	5.21	E4
AX-109485864	2D	643.049151	7.8226E-12	11.11	E1
AX-111461589	2D	645.36891	7.8226E-12	11.11	E1
AX-111956072	2D	34.428838	1.1099E-08	7.95	E4
AX-89644784	2D	73.111103	7.0528E-06	5.15	E4
AX-94576266	2D	646.0323	8.4108E-07	6.08	E1
AX-109911914	3B	701.402417	8.3294E-06	5.08	E1
AX-108891293	3D	418.788268	2.2075E-06	5.66	E4
AX-94465190	3D	603.116286	1.6721E-06	5.78	E1
AX-94748732	3D	570.805113	7.2728E-06	5.14	E1
AX-94536291	3D	571.36	9.09E-05	4.04	BLUP
AX-94942251	3D	569.524232	4.0844E-06	5.39	E4
AX-108858024	4A	237.687734	7.8989E-07	6.10	E1
AX-109306628	4A	88.019454	3.8606E-06	5.41	E2
AX-109306628	4A	88.019454	3.39E-06	5.47	BLUP
AX-109844186	4A	260.186477	7.4415E-12	11.13	E1
AX-109906921	4A	175.850359	4.4213E-08	7.35	E1
AX-110453458	4A	110.280962	4.4504E-11	10.35	E1
AX-109270000	4B	604.866851	7.7292E-06	5.11	E4
AX-109299513	4B	495.595181	4.1025E-11	10.39	E1
AX-109384691	4B	604.924832	7.7292E-06	5.11	E4
AX-109392949	4B	481.507523	1.20E-07	6.92	E4
AX-109392949	4B	481.507523	5.45E-07	6.26	BLUP
AX-110144677	4B	605.156572	6.7405E-06	5.17	E4
AX-111046577	4B	522.801328	5.7912E-06	5.24	E3
AX-111046577	4B	522.801328	8.09E-06	5.09	BLUP
AX-111230804	4B	526.159151	5.7912E-06	5.24	E3
AX-111230804	4B	526.159151	8.09E-06	5.09	BLUP
AX-111649698	4B	609.415296	8.586E-06	5.07	E4
AX-89577308	4B	559.647884	6.1248E-06	5.21	E4
AX-89577308	4B	559.647884	2.43E-05	4.61	BLUP
AX-109916689	5A	10.301112	3.4886E-08	7.46	E1
AX-109934491	5A	618.845609	5.1763E-06	5.29	E2
AX-109934491	5A	618.845609	4.20E-06	5.38	BLUP
AX-110644627	5A	3.555	9.4213E-07	6.03	E4
AX-110644627	5A	3.555	1.80E-07	6.74	BLUP
AX-108803584	5B	707.261636	1.1583E-07	6.94	E1
AX-108829835	6B	151.196592	0.000007297	5.14	E4
AX-110616775	6B	708.659541	9.1494E-06	5.04	E4
AX-110616775	6B	708.659541	1.99E-05	4.70	BLUP
AX-111593090	6B	526.740938	8.4337E-06	5.07	E2
AX-111593090	6B	526.740938	1.11E-05	4.96	BLUP
AX-111028967	6D	29.467167	7.8226E-12	11.11	E1
AX-111931136	6D	222.572988	5.7479E-06	5.24	E1
AX-89742838	7D	199.413838	4.2648E-08	7.37	E4

### Functional prediction of candidate genes for strain height

SNP markers with large phenotypic effect values that could be stably inherited were searched in the Chinese Spring Genome Database of common wheat and BLASTx sequence comparison was performed in the NCBI database, and a total of seven candidate genes most likely to be associated with plant height were mined (**Table 3**). The candidate genes for plant height were mainly related to photosynthesis, potassium transporters, zinc finger family proteins and expressed proteins containing F-box structural domains in crops. Among them, the gene TraesCS1B01G350500 localised on chromosome 1B, encodes a transcription repressor; TraesCS2A01G570600 localised on chromosome 2A, is associated with Agmatine coumaroyltransferase-2; and TraesCS2D01G570600 localised on chromosome 2D, is associated with Agmatine coumaroyltransferase-2. TraesCS2D01G581800 on chro-mosome 2D encodes GRF zinc finger family protein; TraesCS3D01G466000 on chromosome 3D encodes F-box domain containing protein; TraesCS4B01G466000 on chromosome 4B encodes F-box domain containing protein; and TraesCS6D01G061800 on chromosome 6D encodes Potassium transporter.

**Table 3:** Information of candidate genes for wheat plant height.

Marker	Chr.	Position (Mb)	Genes	Gene annotation
*AX-110425403*	1B	581.17	*TraesCS1B01G350500*	transcription repressor
*AX-109472712*	2A	767.94	*TraesCS2A01G570600*	Agmatine coumaroyltransferase-2
*AX-111461589*	2D	645.37	*TraesCS2D01G581800*	GRF zinc finger family protein
*AX-94748732*	3D	570.81	*TraesCS3D01G466000*	F-box domain containing protein
*AX-109299513*	4B	495.6	*TraesCS4B01G238200*	Photosystem I reaction center subunit IV
*AX-110644627*	5A	3.56	*TraesCS5A01G013800*	F-box domain containing protein
*AX-111028967*	6D	29.47	*TraesCS6D01G061800*	Potassium transporter

## Discussion

### Association analysis of plant height

With the rapid development of biology and bioinformatics, GWAS analysis has become an important way to study quantitative traits in plants, and the mining of genes related to wheat plant height has been promoted to a greater extent. Classical genetic studies have shown that plant height is a complex trait jointly controlled by Mendelian and quantitative genes. In this study, multi-environmental genetic locus mining was carried out through four environmental sites, and a total of 44 SNP markers were mined, which were distributed on chromosomes 1B, 1D, 2A, 2B, 2D, 3B, 3D, 4A, 4B, 4A, 6B, 6D, and 7D, with a rate of variation of 5.00-11.11% for the individual inter-pretable phenotypes. In the 1960s, the utilization of dwarf genes such as Rht1 (Reduced Height 1) and Rht2 significantly enhanced wheat’s resistance to stunting and increased yield per unit area. This advancement played a pivotal role in the development of agricultural biotechnology and contributed to the onset of the ‘Green Revolution’ in agricultural production. Among the currently available genes related to the study of wheat plant height, the Rht-B1 and Rht-D1 are located on chromosomes 4B and 4D, respectively, and have been successfully used in wheat breeding worldwide (Xu et al., 2023). In addition to Rht-B1 and Rht-D1 genes (Bazhenov et al., 2015), other PH-related genes such as Rht 8, Rht 13, Rht 22, Rht 24, etc. have also been reported (Divashuk et al., 2012). Many loci were also identified as being located on all chromosomes except chromosomes 3D, 4A, 5D, 7A, 7B and 7D. In addition, the locus AX-109852602 on chromosome 2B mined in this study overlapped with the locus on plant height mined by Li et al. (2022) and the locus AX-111956072 on chromosome 2D overlapped with the locus on plant height mined by Guo et al. (2010).

### Functional analysis of candidate genes

By querying stable inherited SNP markers against the common wheat Chinese Spring genome database, seven candidate genes most likely associated with plant height were identified. These candidate genes are primarily linked to photosynthesis, potassium transporters, zinc finger proteins, and expressed proteins containing the F-box domain. The gene TraesCS1B01G350500 located on chromosome 1B encodes a transcription repressor. Throughout the long evolutionary process, plants have developed intricate gene regulatory networks, where reversible transcription repression and relief responses play a crucial role in plant adaptation to the environment and meeting their own growth and development needs. Genes TraesCS2A01G570600 and Agmatine cou-maroyltransferase-2 associated with chromosome 2A; and TraesCS2D01G581800 encoding GRF zinc finger family protein on chromosome 2D. This protein participates in the physiological and biochemical regulatory mechanisms of plant growth and development. TraesCS3D01G466000 located on chromosome 3D and TraesCS5A01G013800 on chromosome 5A encode F-box domain containing proteins, which are expressed. The F-box protein family plays crucial roles in plant hormone signalling, light signal transduction, and physiological processes such as floral organ development (Kipreos and Pagano, 2000). TraesCS4B01G238200 on chromosome 4B encodes Photosystem I reaction centre subunit IV. Additionally, TraesCS6D01G061800 on chromosome 6D encodes a Potassium transporter. Potassium ions are essential monovalent cations for plant growth and development, being among the most abundant cations in plant cells (Himi et al., 2011). Potassium nutrient uptake is critically important for normal plant growth and development. Potassium ions participate in various processes including enzyme activation, regulation of cell turgor pressure, enhancement of substance synthesis and transport within plants, promotion of photosynthesis, and bolstering crop resistance to diseases and stresses.

## Conclusion

This study utilized a Q+K mixed linear model and employed a 55K SNP chip to conduct a genome-wide association analysis of heading date and maturity in 239 domestic and international wheat varieties. A total of 44 SNP markers significantly associated with wheat plant height were identified (P ≤ 0.00001). These markers were distributed across chromosomes 1B, 1D, 2A, 2B, 2D, 3B, 3D, 4A, 4B, 6B, 6D, and 7D, individually explaining phenotypic variance rates ranging from 5.00% to 11.11%. Notably, in multiple environments, AX-110425403 on chromosome 1B, AX-94748732 on chromosome 3D, and AX-110644627 on chromosome 5A exhibited phenotypic variance rates of 5.00% to 9.68%. Subsequently, SNPs showing substantial phenotypic effects and stable heritability were queried against the common wheat Chinese Spring genomic database, leading to the discovery of 7 promising candidate genes most likely associated with wheat plant height.

## Data Availability

The datasets generated during and/or analyzed during the current study are available from the corresponding author upon reasonable request.
